# Follicular Lymphoma Detected in a Patient Undergoing Mohs Surgery: A Case Report

**DOI:** 10.7759/cureus.77583

**Published:** 2025-01-17

**Authors:** Charlotte Defty, Yamini Krishna, Muhammad Adil A Khan, Naveen Sharma, Hamid Tehrani

**Affiliations:** 1 Plastic and Reconstructive Surgery, St Helens and Knowsley Teaching Hospitals NHS Trust, St Helens, GBR; 2 Cellular Pathology, Liverpool University Hospitals NHS Foundation Trust, Liverpool, GBR; 3 Plastic and Reconstructive Surgery, Northumbria Healthcare NHS Foundation Trust, North Shields, GBR; 4 Histopathology, St Helens and Knowsley Teaching Hospitals NHS Trust, St Helens, GBR

**Keywords:** basal cell carcinoma, cutaneous b cell follicular lymphoma, frozen section, histology, mohs micrographic surgery

## Abstract

Mohs surgery is performed by surgeons trained in detecting specific cutaneous malignancies, including basal cell carcinoma (BCC), squamous cell carcinoma (SCC), and dermatofibrosarcoma protuberans (DFSP). Mohs-trained surgeons are typically not holistic pathologists and may, when working in isolation, fail to identify lesions that are not of cutaneous origin. The presence of a second lesion adjacent to the primary lesion is always a possibility. Such a lesion may be misinterpreted as an extension of the identified primary lesion, leading to unnecessary surgery, or it may be overlooked, resulting in a diagnostic failure. There is an advantage to conducting Mohs surgery with the surgeon and histopathologist reviewing the frozen section slides together, as this approach can aid in the identification of rarer diagnoses. Here, we describe the case of a female patient who underwent Mohs micrographic surgery (MMS) for a recurrent BCC located in the left preauricular area and medial helical rim of the pinna. The surgery involved two excisional stages. The first stage showed morphoeic BCC at all levels of all blocks. The second stage showed no BCC but revealed a dense inflammatory infiltrate. On further assessment by the consultant histopathologist, this infiltrate raised suspicion of possible lymphoma. Based on this consensus, no further Mohs excisional surgery was performed, and the surgical defect was closed. Formalin-fixed paraffin-embedded (FFPE) histological assessment and immunohistochemistry confirmed the diagnosis of cutaneous B-cell follicular lymphoma (FL). Thus, the incidental finding on the examination of fresh frozen MMS sections was correctly interpreted by the combined approach of the Mohs surgeon and pathologist, guiding appropriate and timely management for the patient.

## Introduction

Mohs micrographic surgery (MMS) is a specialized skin cancer treatment pioneered by Dr. Frederick Mohs in the 1930s at the University of Wisconsin. Over time, MMS has gained widespread use in the dermatologic surgery community and is now considered the treatment of choice for many common and rare cutaneous neoplasms [1].

Skin cancers often have small root-like extensions that can be missed when excised tumors are serially cross-sectioned in a bread-loaf fashion, a method commonly used for excision specimens. Hence, traditional surgery compensates for this by employing wider surgical margins. MMS allows for the excision of skin tumors with minimal margins, sparing healthy tissue, while providing complete histopathological analysis of both the peripheral and deep surgical margins. This results in higher cure rates compared to traditional surgery [1].

Based on tumor characteristics, anatomic location, and unique patient characteristics, tumors are classified as low- or high-risk tumors. High-risk BCC, high-risk SCC, and rare non-melanoma skin cancers (NMSCs) (e.g., dermatofibrosarcoma protuberans (DFSP), mucinous carcinoma, atypical fibroxanthoma, sebaceous carcinoma, microcystic adnexal carcinoma, and extramammary Paget's disease) should be referred for MMS. Additionally, melanoma in situ (MIS) in high-risk areas [2] and even invasive melanomas are increasingly being treated with MMS, demonstrating cure rates comparable to or often better than wide local excision (WLE) [3,4].

MMS is a safe procedure with very few complications, most of which are managed by Mohs surgeons in their offices. MMS is traditionally performed under local anesthesia and only rarely under conscious sedation or general anesthesia [5]. Patients who may be poor candidates for general anesthesia in the operating room can usually undergo MMS. A large, 23-center prospective study reported minor and serious postoperative complications associated with MMS at 0.72% and 0.02%, respectively. Among the serious complications, none occurred during surgery; they were mostly related to local infection in the postoperative period. All hospitalized patients recovered successfully with IV antibiotics, and no life-threatening events or deaths occurred [5].

Mohs surgeons receive intensive training in cutaneous surgery, frozen section pathology, and surgical defect reconstruction after complete excision of skin neoplasms. However, they are not holistic pathologists and may overlook lesions not of cutaneous origin when working in isolation. There is an advantage to conducting Mohs surgery with the surgeon and histopathologist reviewing the frozen section slides together, as this collaborative approach can help identify rarer lesions that might otherwise be missed, especially when they coexist with common lesions like BCC.

## Case presentation

**Figure 1:**
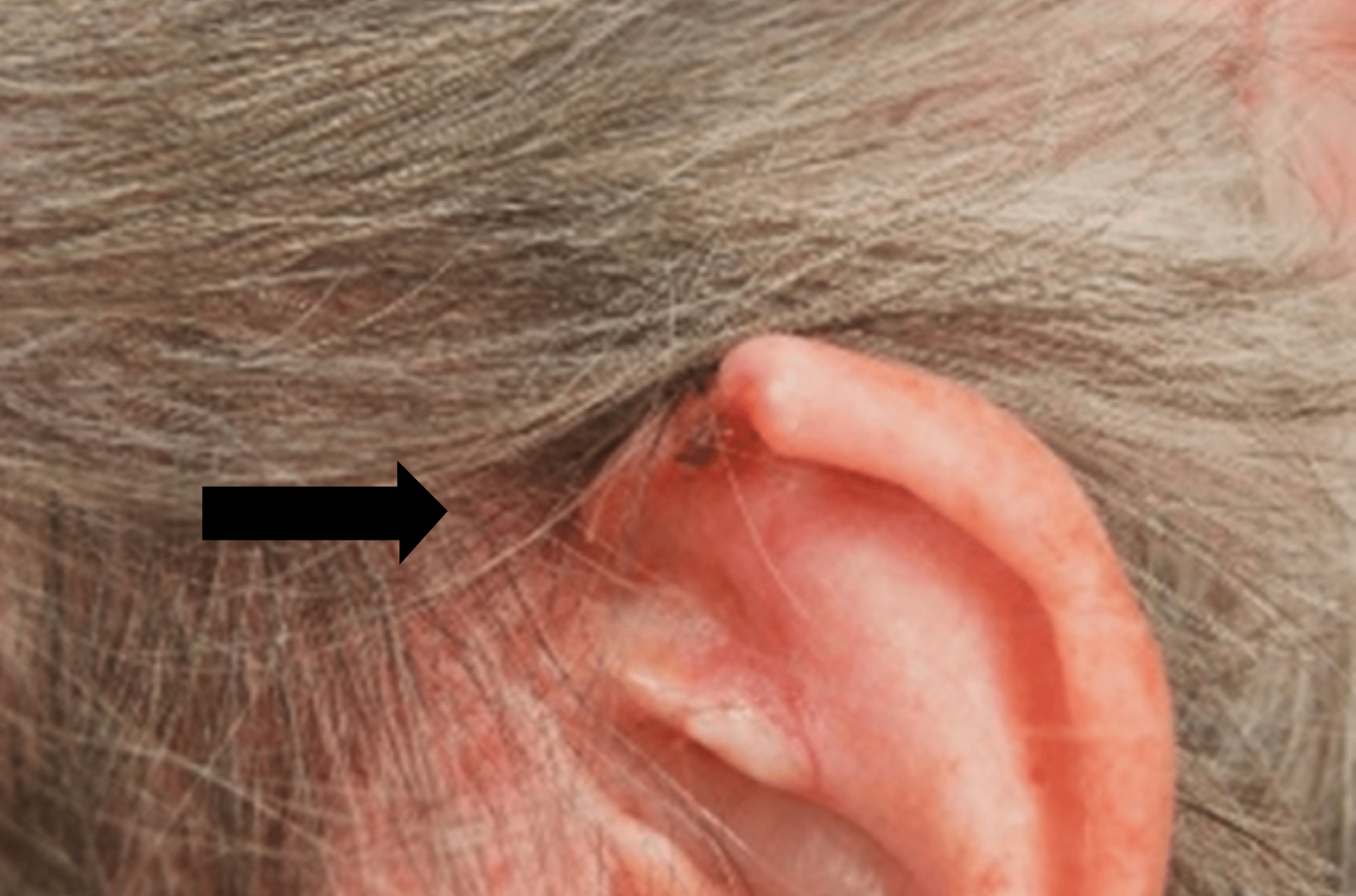
Photo of the recurrent BCC on the left preauricular area and medial helical rim of the pinna BCC: basal cell carcinoma.

An 84-year-old female patient was referred to our plastic surgery department for MMS due to a recurrent, biopsy-confirmed infiltrative BCC located in the left preauricular area and medial helical rim of the pinna (Figure 1). 

The patient had initially undergone conventional excision of a lesion from the same area two years earlier. Histopathological analysis at that time confirmed complete excision of a nodular and infiltrative BCC, with a deep margin clearance of 1.5 mm and a peripheral margin clearance of 4 mm. No evidence of perineural invasion was observed.

**Figure 2:**
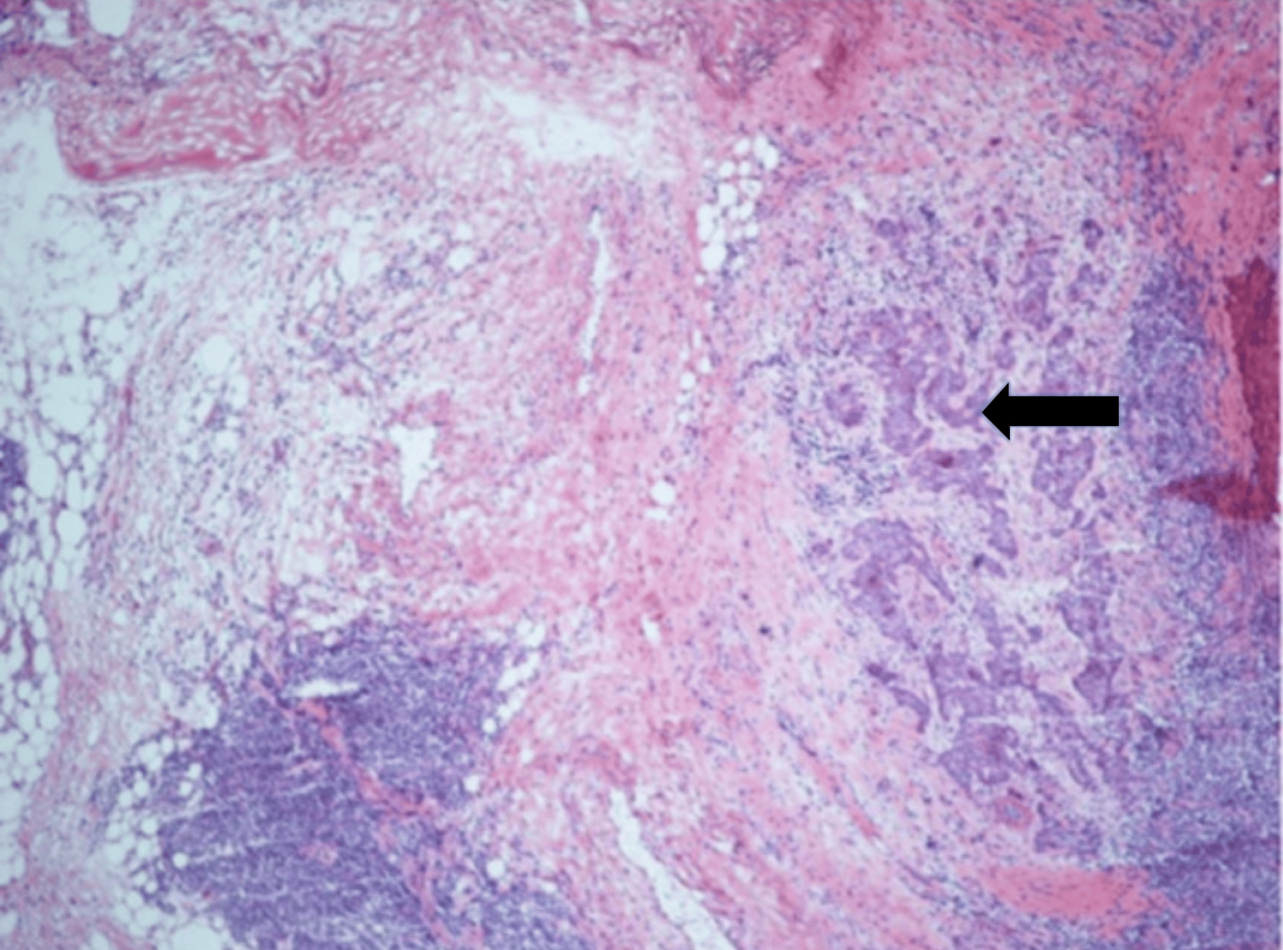
Frozen section from stage I of the Mohs excision showing morphoeic BCC (arrow) with dense lymphoid infiltrate (H&E x40 magnification) later confirmed as lymphoma BCC: basal cell carcinoma.

The subsequent Mohs surgery involved two excisional stages, with a total of 12 tissue blocks. The first stage showed morphoeic BCC at all levels of all blocks (Figure 2). 

**Figure 3:**
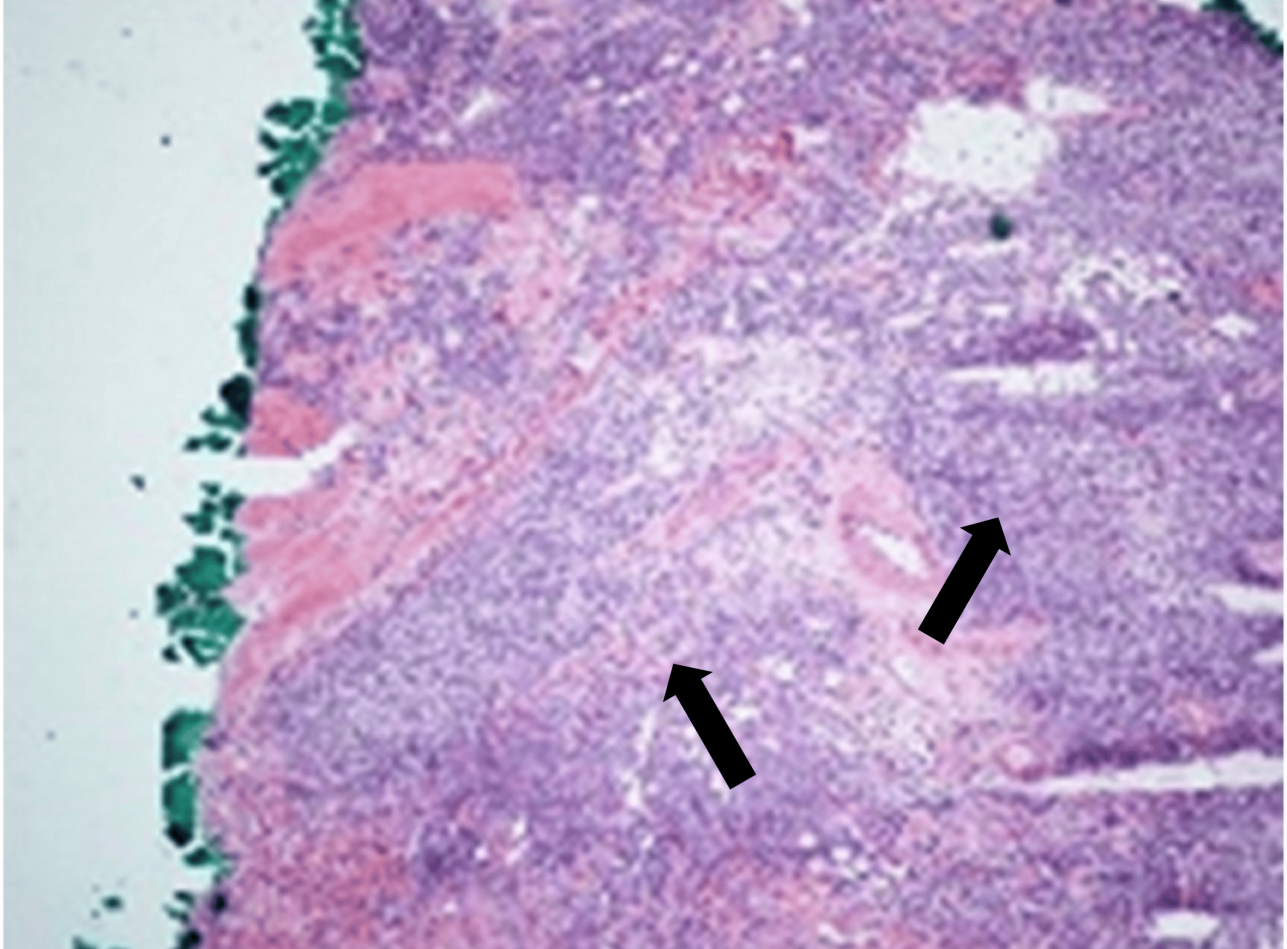
Persistent widespread lymphocytic inflammation seen on frozen sections from stage II Mohs excision (H&E x40 magnification)

The second stage was free of BCC but showed further dense inflammatory infiltrate. While an inflammatory infiltrate around BCC is a common feature, the pathologist's expert evaluation identified a dense infiltrate with an irregular nodular pattern. This infiltrate consisted of a mixture of centrocytes (small germinal center B lymphocytes with cleaved nuclei) and larger centroblasts (activated germinal center B lymphocytes with large vesicular nuclei and 2-3 nucleoli adjacent to the nuclear membrane), raising suspicion of possible lymphoma (Figure 3).

No further Mohs excisional surgery was performed, and the tissue blocks were formalin-fixed paraffin-embedded (FFPE) for further histological analysis.

**Figure 4:**
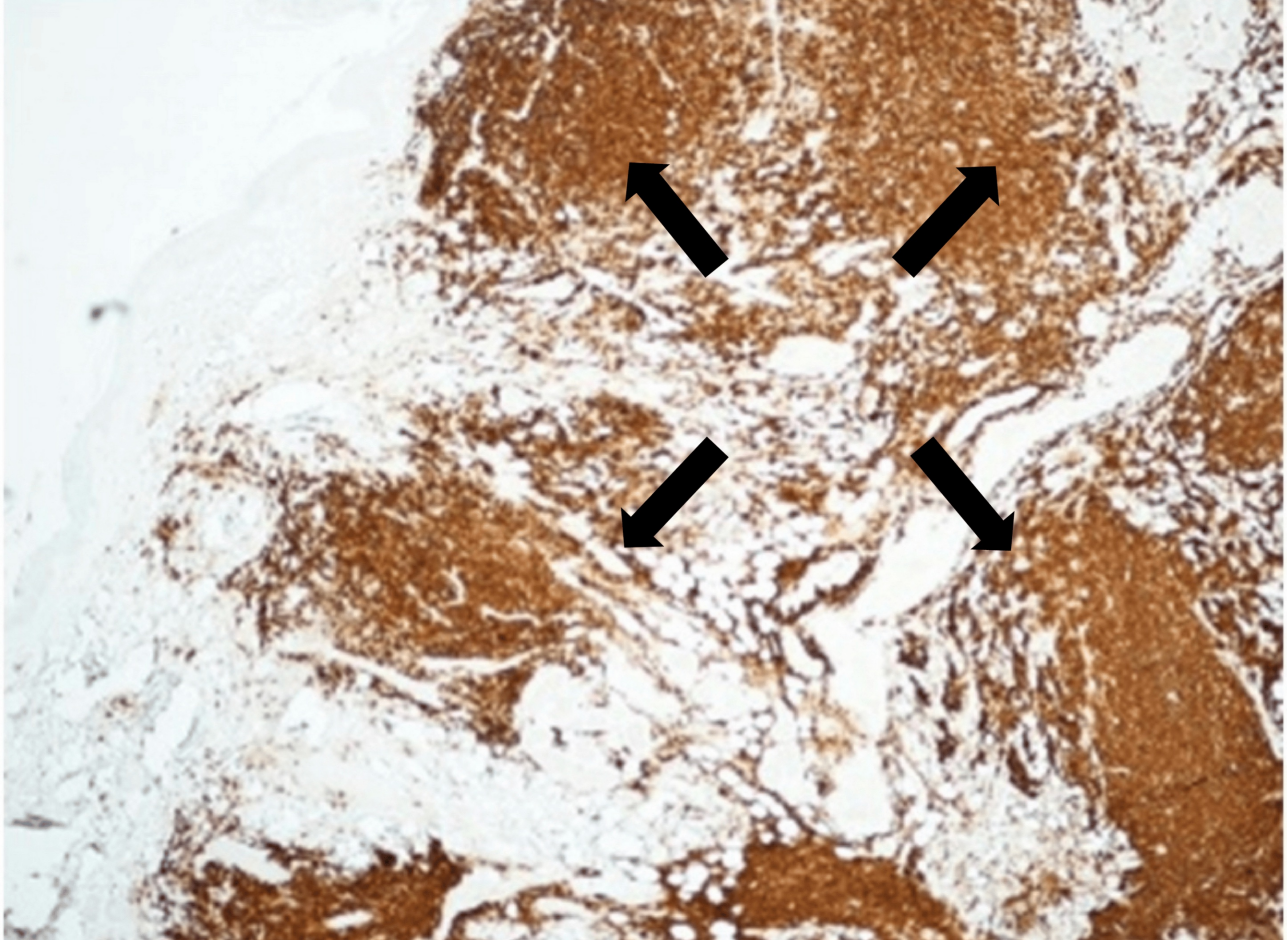
Immunohistochemistry showing positive CD20 immunostaining highlighting the B-cell lineage of lymphocytes (3,3'-diaminobenzidine (DAB) x40 magnification)

The patient had not undergone ear reconstruction during the initial surgery and declined any further surgical or prosthetic options at this time as well. The excised edge of the pinna was therefore closed directly, and a full-thickness skin graft was used for the preauricular defect. Histological assessment revealed a dense lymphoid infiltrate with an irregular nodular pattern, which was distant from the BCC, and composed of small and medium-sized lymphoid cells, along with occasional larger blasts. The nodules were present throughout the dermis, some near skin appendages, and importantly extending into subcutaneous fat. The neoplastic cells were strongly positive for B-cell markers (CD20 and CD79a) (Figure 4) and germinal center markers (CD10, BCL2, and BCL6), while negative for cyclin D1. Residual follicular meshwork cells were highlighted by CD23. Background reactive T-cells (CD3+ and CD5+) were also noted. These findings were highly suspicious for cutaneous B-cell follicular lymphoma (FL), prompting referral to the Haemato-Oncology Diagnostics System (HODS; Royal Liverpool University Hospital) for specialist evaluation. Further biopsies have since been performed on other lesions on the patient’s facial area with similar histological findings. The polymerase chain reaction (PCR) analysis for B-cell receptor gene rearrangements detected a monoclonal B-cell expansion, and the final report from HODS confirmed a diagnosis of grade 1-2 follicular B-cell lymphoma, which had spread from the lymph nodes to the skin. The presence of nodal lymphoma was not known until the suspicion was raised by the histopathologist during Mohs surgery.

## Discussion

This case highlights the importance of close collaboration between the surgeon and histopathologist to ensure an accurate diagnosis and prompt management of the patient.

FL accounts for 10%-20% of all lymphomas, with the highest prevalence in the USA and Western Europe [6]. It mainly affects adults (median age of 65 years) with a slight male preponderance, although no sex predilection has been observed [7,8]. FL predominantly affects the lymph nodes, but extranodal involvement is very common, and most patients present with widespread disease at diagnosis [9-11]. In this case, FL had spread from the lymph nodes to the skin, but the presence of nodal lymphoma was unknown until the suspicion was raised during Mohs surgery.

FL is a neoplasm of germinal center B-cells, with varying proportions of centrocytes and centroblasts, with at least a partially follicular growth pattern. In rare cases where a diffuse growth pattern is observed, the tumor cells still show germinal center B-cell morphology and immunophenotype [12].

Around 10%-15% of patients present with stage I or II. Most are asymptomatic, and B-symptoms (e.g., fever, night sweats, and weight loss) are uncommon. Lesions may wax and wane without therapy, and the clinical course takes a chronic, relapsing pattern, complicating both diagnosis and treatment.

FL is an indolent disease with a median survival of >17 years with treatment [13,14]. Its prognosis is closely related to the extent of disease at diagnosis, as well as the occurrence of certain mutations. Mutations in the EZH2 gene tend to confer a more favorable response to immunochemotherapy, while TP53 mutations and “double hit” BCL2 and MYC rearrangements are associated with a poorer prognosis [15-17]. Staging is performed using the Lugano classification. Treatment options for FL depend on the subtype and grade, staging, and the patient’s overall health. These options include immunotherapy, immune-chemotherapy, and radiotherapy (only for early-stage/localized disease). Early treatment does not seem to prolong survival [13,14].

## Conclusions

In the presented case, cutaneous lymphoma was an incidental finding during the examination of fresh frozen MMS sections, identified early through the combined consensus of the Mohs surgeon and pathologist. This helped avoid further unnecessary surgery and allowed for timely and appropriate treatment. Cutaneous lymphoma can mimic the inflammatory infiltrate surrounding BCC on a frozen section, and we hope this case raises awareness among Mohs surgeons. Such awareness would aid in the early identification of lymphoma. Our case also highlights the benefit of collaboration between the Mohs surgeon and pathologist for improved diagnosis and serves as a valuable lesson that the presence of a common lesion should not overshadow the possibility of a coexisting rarer condition.
